# Parechovirus A Circulation and Testing Capacities in Europe, 2015–2021

**DOI:** 10.3201/eid3002.230647

**Published:** 2024-02

**Authors:** Laura Bubba, Eeva K. Broberg, Thea K. Fischer, Peter Simmonds, Heli Harvala, European Non-polio Enterovirus Network working group

**Affiliations:** European Non-Polio Enterovirus Network, Geneva, Switzerland (L. Bubba);; European Centre for Disease Prevention and Control, Solna, Sweden (E.K. Broberg);; University Hospital of Nordsjaelland Department of Clinical Research, Hilleroed, Denmark (T.K. Fischer);; University of Copenhagen Department of Public Health, Copenhagen, Denmark (T.K. Fischer);; University of Oxford Nuffield Department of Medicine, Oxford, UK (P. Simmonds);; National Health Service Blood and Transplant, London, UK (H. Harvala);; University College London Division of Infection and Immunity, London (H. Harvala)

**Keywords:** parechovirus, viruses, enteric infections, meningitis/encephalitis, surveys, epidemiology, circulation, seasonality, PeV-A3, PeV-A4, PeV-A5, severity, Europe

## Abstract

Parechovirus infections usually affect neonates and young children; manifestations vary from asymptomatic to life-threatening. We describe laboratory capacity in Europe for assessing parechovirus circulation, seasonality, and epidemiology. We used retrospective anonymized data collected from parechovirus infection case-patients identified in Europe during January 2015–December 2021. Of 21 laboratories from 18 countries that participated in the study, 16 (76%) laboratories with parechovirus detection capacity reported 1,845 positive samples; 12/16 (75%) with typing capability successfully identified 517 samples. Parechovirus A3 was the most common type (n = 278), followed by A1 (153), A6 (50), A4 (13), A5 (22), and A14 (1). Clinical data from 1,269 participants highlighted correlation of types A3, A4, and A5 with severe disease in neonates. We observed a wide capacity in Europe to detect, type, and analyze parechovirus data. To enhance surveillance and response for PeV outbreaks, sharing typing protocols and data on parechovirus-positive cases should be encouraged.

Parechoviruses are small, nonenveloped, single-stranded RNA viruses belonging to the large Picornaviridae family that circulate worldwide; primary infections occur mainly in children <2 years of age (*1*,*2*). Parechoviruses are transmitted by fecal-oral and respiratory routes (*2*,*3*). Most infections are asymptomatic or have mild general gastrointestinal or respiratory symptoms, but they can occasionally lead to sepsis, meningitis or other neurologic manifestations, or even death (*2*–*6*). 

Nineteen human parechovirus types have been classified as species types PeV-A1–A19 (*7*); the most commonly reported are A1, A3, and A6 (*2*,*3*). PeV-A1 and A6 infections are generally associated with mild outcomes, but PeV-A3 can cause severe neurologic disease in infants <3 months of age (*2*,*4*–*6*,*8*,*9*). More recently, PeV-A4 and A5 also have been associated with severe clinical manifestations in children (*10*,*11*). Recorded genotype distribution might vary on the basis of study design, including testing strategy, geographic location, and timing of sampling, because epidemiology can differ by virus type (*3*). Data collected from nonpolio enterovirus (NPEV) surveillance and childhood prevalence studies showed worldwide parechovirus distribution differs by genotype; PeV-A1 is the most prevalent type in the United States, Asia, and Europe, followed by A3 and A4 (*12*). PeV-A6 is reported as second most common in Australia and in some studies in Europe (*2*,*12*). Additional genotypes, including A2 and A7–A19, that are rare in Europe and the United States have been reported in India, Pakistan, and Africa (*12*). 

Parechovirus studies in Europe have mostly focused on children or specific symptoms, with no data from dedicated surveillance and limited data from the NPEV surveillance system. The lack of systematically collected data limits full understanding of the impact and circulation of parechovirus infections. Clarifying the epidemiology, clinical implications, and phylogeny of parechovirus would help laboratories and national health authorities make decisions about the clinical relevance of infections. We therefore conducted a retrospective study to assess the presence of surveillance and laboratory capacity for parechovirus detection and typing in Europe during 2015–2021. We also described the seasonality, clinical manifestations, and molecular epidemiology of parechovirus infections during the 7-year study period (2015–2021). 

## Methods

### Data Collection

In March 2022, the European Non-polio Enterovirus Network (ENPEN) invited the national focal point agencies that constitute the European Centre for Disease Prevention and Control (ECDC) public health network, regional reference laboratories from all 30 member states within the European Union (EU), European Economic Area, the United Kingdom, and local laboratories affiliated with ENPEN to join the study. We sent a reminder letter about participation 15 days before the deadline. 

We used data collected during January 1, 2015–December 31, 2021 as part of an EU survey (*13*). The survey included questions for each participating laboratory on their extent of and approach to parechovirus detection and surveillance and their screening policies and capacity for detection and typing. We also requested information on methods used in each laboratory (Appendix Table 1). When available, we collected anonymized aggregated data on monthly and yearly parechovirus detection, associated clinical symptoms, age group, sample type, sex, and total number of samples tested for each study year by parechovirus type (Appendix Figure 1). Each laboratory collected data from various sources, such as NPEV, acute flaccid paralysis, and influenza-like illness (ILI) surveillance; screening of hospital admissions records; and cerebrospinal fluid (CSF) samples. 

For each laboratory we summarized the capacity for parechovirus detection and what triggers they used to initiate testing (Table). We included laboratories reporting the absence of parechovirus testing, to better understand the extent of testing capacity in Europe. We asked participating laboratories to share nucleotide sequences of PeV-A3 strains that had been typed; in cases of outbreaks or clusters, we requested only nonidentical (i.e., differing by ≥2%) sequences. 

**Table:** Information about institutions included in study of parechovirus testing, surveillance, and genotyping capacities, Europe*

Country (region)†	Institution type	PeV-A testing capacity	Timeframe of data	National surveillance	Testing trigger	Genotyping capacity	Routine genotyping
Austria	Hospital virology or microbiology laboratory	Yes	2015–2021	No	Upon clinical request; all CSF from children <12 mo sent to institute tested for PeV-A	Yes	No
Denmark	National PH institute	Yes	2015–2021	Yes	Surveillance system: positive samples from clinical microbiology laboratories after clinical request forwarded to SSI for sequencing	Yes	Yes
Finland	Hospital virology/ microbiology laboratory	Yes	2015–2021	Yes‡	Upon clinical request	Yes	No
Ireland	Diagnostic and virus reference laboratory	Yes	2015–2021	No	Passive surveillance system triggered by clinical request. PeV-A testing on all CSF from CNS virology screen from children <3 y	No	NA
Italy (Lombardy)	Regional PH institute and academic institution	Yes	2015–2021	No§	ILI and AFP surveillance, clinical requests	Yes	Yes
Italy (Lombardy)	Hospital virology/ microbiology laboratory and academic institution	Yes	2015–2021	No	Hospital request for PeV-A testing in patients with clinical manifestations of meningo-encephalitis, encephalitis, or sepsis	Yes	Yes
Italy (Lombardy)	Hospital microbiology	Yes	2015–2021	No	Upon clinical request	No¶	NA
Slovenia	Academia	Yes	2015–2021	No	Upon clinical request	Yes	No
Spain	National PH institute	Yes	2015–2021	Yes‡	No specific criteria; Nacional Centre for Microbiology’s Enterovirus and Parechovirus Reference Lab receives 500–700/y EV- and PeV-A-positive samples voluntarily submitted for genotyping and 20–30/y samples for EV/PeV testing	Yes	Yes
UK (England)	National PH institute	Yes	2015–2021	Yes‡	Samples from non-polio EV passive surveillance system. Voluntarily submitted samples PeV-A-positive samples for confirmation and genotyping. (voluntary because PeV-A is not a notifiable pathogen)	Yes	No
Netherlands	Hospital virology/ microbiology laboratory and academic institution	Yes	2015–2021	Yes‡	Samples from the non-polio EV passive surveillance system	Yes	Yes
Norway	National PH institute	Yes	2015–2017	No	Upon clinical request	No	No#
UK (Scotland)	National PH institute	Yes	2015–2017	No	Upon clinical request	Yes	No#
Luxembourg	National PH institute	Yes	2015–2021	No	CSF from clinical requests	No	NA
Slovenia	National PH institute	Yes	2015–2021	No§	ILI surveillance system	No	NA
Poland	National PH institute	Yes	2015–2021	No	Upon clinical request	Yes	No
Bulgaria	National PH institute	Not yet performed	No data reported	Not yet performed	Not yet implemented	NA	NA
Czechia	National PH institute	No	No data reported	No	None	NA	NA
Estonia	National PH institute	No	No data reported	No	None	NA	NA
Hungary	National PH institute	No	No data reported	Unknown	None	NA	NA
Slovak Republic	National PH institute	No	No data reported	No	None	NA	NA

*ACP, acute flaccid paralysis; CNS, central nervous system; CSF, cerebrospinal fluid; EV, enteroviruses; PeV-A, parechovirus type A; ILI, influenza-like illness; PH, public health; SSI, Statens Serum Institut. †Countries of origin of the participating laboratories. ‡Implemented in the passive non-polio enterovirus surveillance. §All respiratory samples collected for the influenza-like illness surveillance were screened for parechoviruses. ¶Parechovirus-positive samples were sent to the regional reference laboratory for sequencing. #All samples included in the acute flaccid paralysis (AFP) surveillance are also screened for parechovirus.

### Data Analysis

We reported the number of parechovirus infections by month/year and country of study, and analyzed data by clinical symptoms, age group, sample type, and parechovirus type when information was available. We calculated overall parechovirus detection rate when total number of samples tested was reported. Because some laboratories did not implement parechovirus detection testing until after the study had begun, we reported proportions of positive samples for the entire 2015–2021 study period and for the specific timeframes 2015–2017 and 2018–2021. We calculated parechovirus type distribution by year, clinical symptoms, age group, sample type, and month, and calculated the proportion of detections and types of samples. We performed χ^2^ testing using Vassar stat (*14*) to compare proportions; p<0.05 indicates statistical significance. 

For PeV-A3 analysis, we summarized 106 sequences with >80% completeness in viral protein (VP) 3/VP1 junction region positions 2182–2437 (as numbered in the echovirus 22 prototype sequence L02971) (Appendix Table 2). We aligned sequences using MUSCLE 3 (*15*) and compared them with 630 publicly available PeV-A3 nucleotide sequences from this region retrieved from GenBank database in December 2022 using sequence editor version 1.4 (*16*). In addition, participating laboratories provided 30 sequences from a second region in VP1 (positions 2336–3038; Appendix Table 2), which we compared with 856 available GenBank sequences. We performed neighbor-joining phylogenetic analysis (Jukes-Cantor model) and calculated maximum likelihood using the optimal substitution model, Tamura-Nei with γ correction, using MEGA package version 7 (*17*). When sampling dates were available, we inspected phylogenetic trees for country-specific clustering and temporal trends. 

## Results 

In total, 21 laboratories from 18 EU and European Economic Area member states participated in the study; 16/21 participating laboratories performed parechovirus testing (76%). Of those not testing, 1 laboratory each in the Slovak Republic and Bulgaria planned to introduce parechovirus in routine diagnostics, but the remaining 3 laboratories, in the Czech Republic, Estonia, and Hungary, had no plans to implement nationwide parechovirus testing (Table). Of the 16 laboratories performing testing, 11 (69%) provided data for 2015–2021; 2, in Norway and the United Kingdom (Scotland), provided data only for 2015–2017, and 3, in Luxemburg, Poland, and Slovenia, reported data for 2017–2021 after commencing testing. 

Twelve (75%) of 16 laboratories initially performing testing reported capacity to type parechovirus-positive samples and provided type information for this study (Table). Of those, 5 performed sequencing routinely and 7 sequenced viruses only from selected clinically detected cases. Most (11/12) laboratories analyzed sequences in the VP3/VP1 junction region positions 2182–2437, but 1 laboratory, in the Netherlands, performed sequencing from the start of VP1 (positions 2336–3038). To perform the analysis of this region, we alternatively used data from Denmark, Poland, and the United Kingdom (England) because they provided data from a longer portion of the parechovirus genome that included VP1 (Appendix Table 2). 

### Parechovirus Detection 

**Figure 1:**
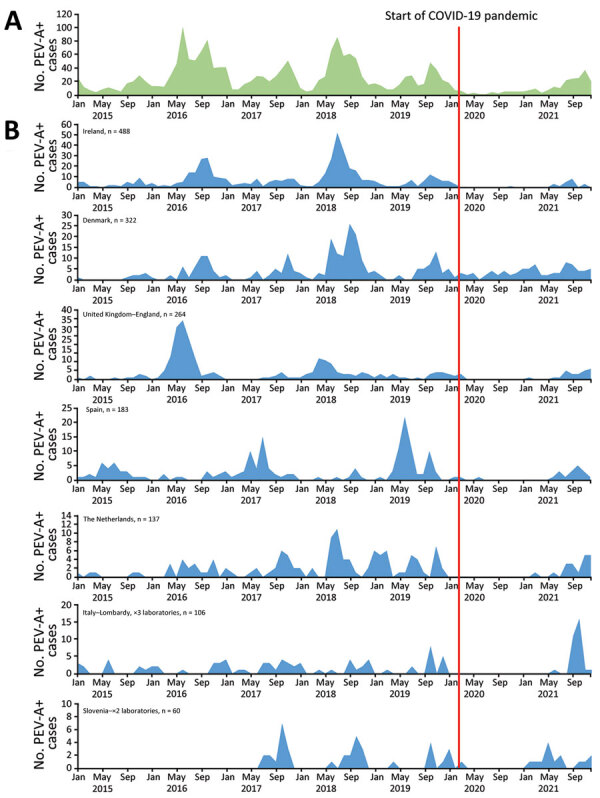
Monthly distribution of parechovirus in Europe, overall and by country, 2015–2021. A) Overall monthly distribution. B) Monthly distribution for countries reporting >50 infections.

Sixteen laboratories from 13 countries reported 1,845 parechovirus-positive samples. Finland, the Netherlands, Spain, and England added parechovirus data based on voluntarily reporting positive cases to the national laboratory, to existing enterovirus surveillance (Table). Those 4 countries reported the most (65%, n = 1,200) parechovirus-positive samples. One laboratory each in Slovenia and in the Lombardy region of Italy (Italy/Lombardy) that introduced parechovirus screening into ILI surveillance provided ≈130 parechovirus-positive respiratory samples. The same laboratory in Italy/Lombardy detected parechovirus-positive samples from cases identified through the Acute Flaccid Paralysis Surveillance network, which routinely screens for polioviruses. Remaining cases were identified after clinician requests for testing not based on existing NPEV, ILI, or other surveillance systems (Table). Ireland reported the highest number of parechovirus-positive samples (26%, n = 488), followed by Denmark (17%, n = 322) and England (14%, n = 264) (Figure 1). Unfortunately, those countries provided no denominator information, so we could not calculate positivity rates. 

Parechovirus testing capacity, measured by samples tested in 9 laboratories (3 in Italy and 1 each in Austria, Finland, Luxemburg, Poland, Slovenia, and the Netherlands), increased from 8,665 during 2015–2017 to 14,263 during 2018–2021; those laboratories reported 309 positive samples, 100 in 2015–2017 and 209 in 2018–2021. Although parechovirus-positive samples increased over that time, parechovirus detections per number of screened samples remained unchanged: 100/8,665 (1.3%) during 2015–2017 and 209/14,263 (1.5%) during 2018–2021. Detection rate for the entire 2015–2021 study period was 1.4% (309/22,928). 

### Seasonality

All participating laboratories reported month and year of collection of parechovirus-positive samples (Table; Figure 1). Infections were reported every year; 2016 accounting for 24% and 2018 for 25% of detections. Most cases were detected during June–November each year. 

### Distribution of Parechovirus Types 

**Figure 2:**
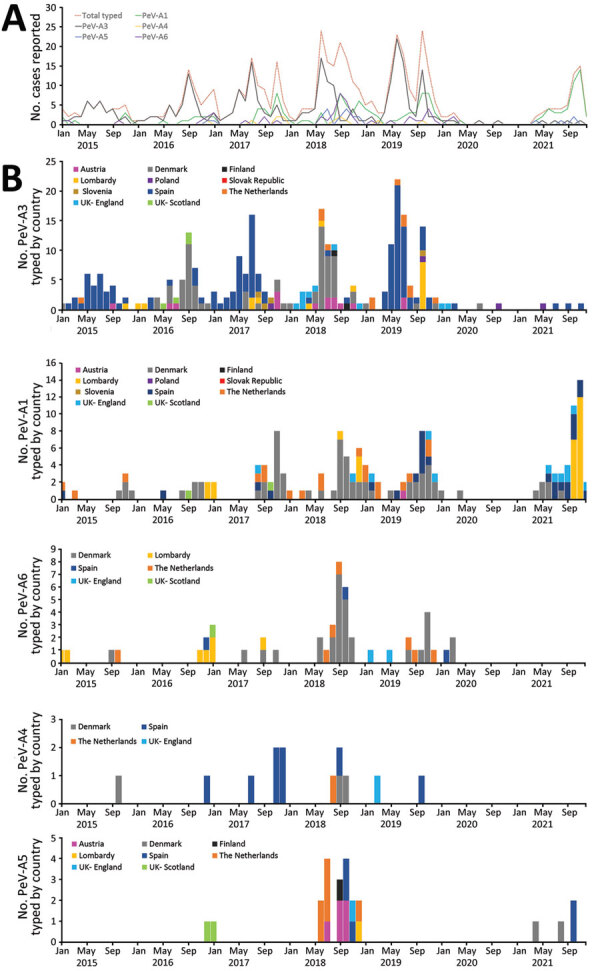
Monthly distribution of typed parechoviruses in Europe, by type and country, 2015–2021. A) Overall monthly distribution. B) Monthly distribution of each detected type by country of detection. Poland reported 1 type A14 infection in June 2021 (not shown).

Twelve laboratories, 10 of which supplied data for the whole study period (Table), reported 517 (45%) of the 1,139 successfully sequenced parechovirus-positive samples, corresponding to 28% (517 /1,845) of all positive samples reported in this study. Among 6 parechovirus types detected, PeV-A3 (54%, n = 278) was the most frequently reported, followed by A1 (30%, n = 153), A6 (10%, n = 50), A5 (4%, n = 22), A4 (2%, n = 13), and A14 (0.2%, n = 1) (Figure 2). Positive PeV-A1 and A3 samples were reported each year during 2015–2021. PeV-A3 accounted for most typed samples in 5/7 study years: 71% in 2015, 75% in 2016, 61% in 2017, 61% in 2019, and 50% in 2020; A5 accounted for 23/31 (74%) of typed samples in 2018 and A1 for 44/52 (85%) in 2021. 

### Geographic Distribution of Parechovirus Types

Spain (35%) and Denmark (33%) provided the most parechovirus case reports with typing information (Figure 2). All laboratories performing typing reported PeV-A3 cases, the most being from Spain (n = 138), Denmark (72), and Italy/Lombardy (17). PeV-A3 exhibits a biannual cycle; most parechovirus cases reported by Denmark were identified in even years (2016 and 2018), whereas most cases reported by Spain occurred in uneven years (2015, 2017, and 2019). Denmark and the Netherlands reported the most PeV-A1 and A6 cases; the Netherlands (35.3%, n = 6), Austria (29.4%, n = 5), and Spain (17.6%, n = 3) reported the most A5 cases. Spain reported 8/13 (62%) A4 cases and Poland reported 1 A14 case. 

### Sample Types 

Sample type information was available for 1,294 positive samples from 13 laboratories. Fecal (n = 447; 35%), CSF (391; 30%), and respiratory (259; 20%) specimens were the sample types most often collected for parechovirus testing; in some cases patients might have provided >1 sample type for testing. CSF was the most common specimen type collected in Austria, Luxemburg, Spain, England, and Scotland; feces in Denmark, Ireland, and the Netherlands; and respiratory specimens in Italy/Lombardy and Slovenia. 

From the 136 successfully typed CSF samples, PeV-A3 (40%), A4 (44%), and A5 (22.7%) were the only types reported, whereas PeV-A1 (50%), A6 (41%), and A5 (52%) were identified from 208 fecal samples. From the 90 respiratory samples typed, PeV-A1 (61%) was the most commonly reported, followed by A3 (20%), A6 (12%), and A5 (7%); no type A4 was reported in respiratory samples.

### Demographic Information and Clinical Manifestations 

Demographic information was available for 1,299 and clinical information for 1,269 parechovirus case-patients reported from 14 laboratories in 11 countries. Male patients (61%, n = 763) and infants <1 year of age (76%, n = 987) accounted for most reported cases; infants <3 months of age accounted for 777 (60%) of reported cases. Symptoms were reported for 1,232/1,479 (83%) cases; fever (23%, n = 305) and neurologic signs (21%, n = 280) were the most common, followed by respiratory symptoms (13%, n = 170). Among patients with less common signs and symptoms, 45 (3.4%) children manifested sepsis, 2 were diagnosed with cardiomyopathy, and 1 with hepatitis. Three children diagnosed with PeV-A1 infection in the Netherlands in 2017 died, but it is unknown whether death was related to parechovirus infection. 

**Figure 3:**
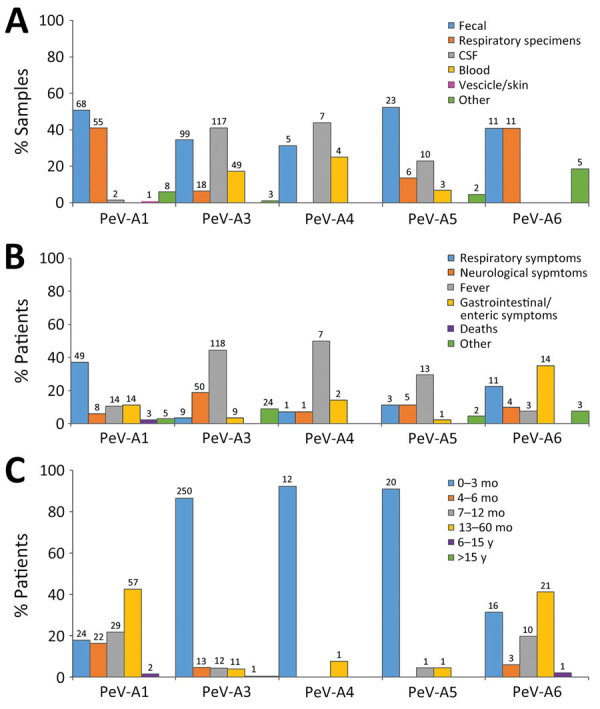
Detection frequencies of different parechovirus types in Europe, 2015–2021, by sample type (A), disease manifestation (B), and patient age (C). Numbers above bars indicate numbers of cases. CSF, cerebrospinal fluid; PeV, parechovirus type.

Information on age groups and symptoms were available for 509/518 (98%) successfully typed cases. The most-reported symptom was fever in children infected with PeV-A3 (44%), A4 (50%), and A5 (30%); among children infected with PeV-A6, gastrointestinal (35%) and respiratory (25%) symptoms were the most commonly reported. Respiratory symptoms (37%) were also common among children infected with PeV-A1 (Figure 3). Most children infected with PeV-A3 (87%), A4 (92%), and A5 (91%) were <3 months of age, whereas >82% of children infected with PeV-A1 were >3 months of age (p<0.0001). Parechovirus infections were rare (n = 68) in children and persons >15 years of age; in that age range, only 1/68 viruses was successful typed and identified as PeV-A3. All detected parechovirus types were associated with neurologic symptoms, of which 72% were typed as PeV-A3, followed by A1 (11%), A5 (7%), A6 (6%), and A4 (1%). The sole PeV-A14 case was detected in a fecal specimen collected from a child with neurologic symptoms from the 6–15-year age group. 

### Phylogenetic Analysis

**Figure 4:**
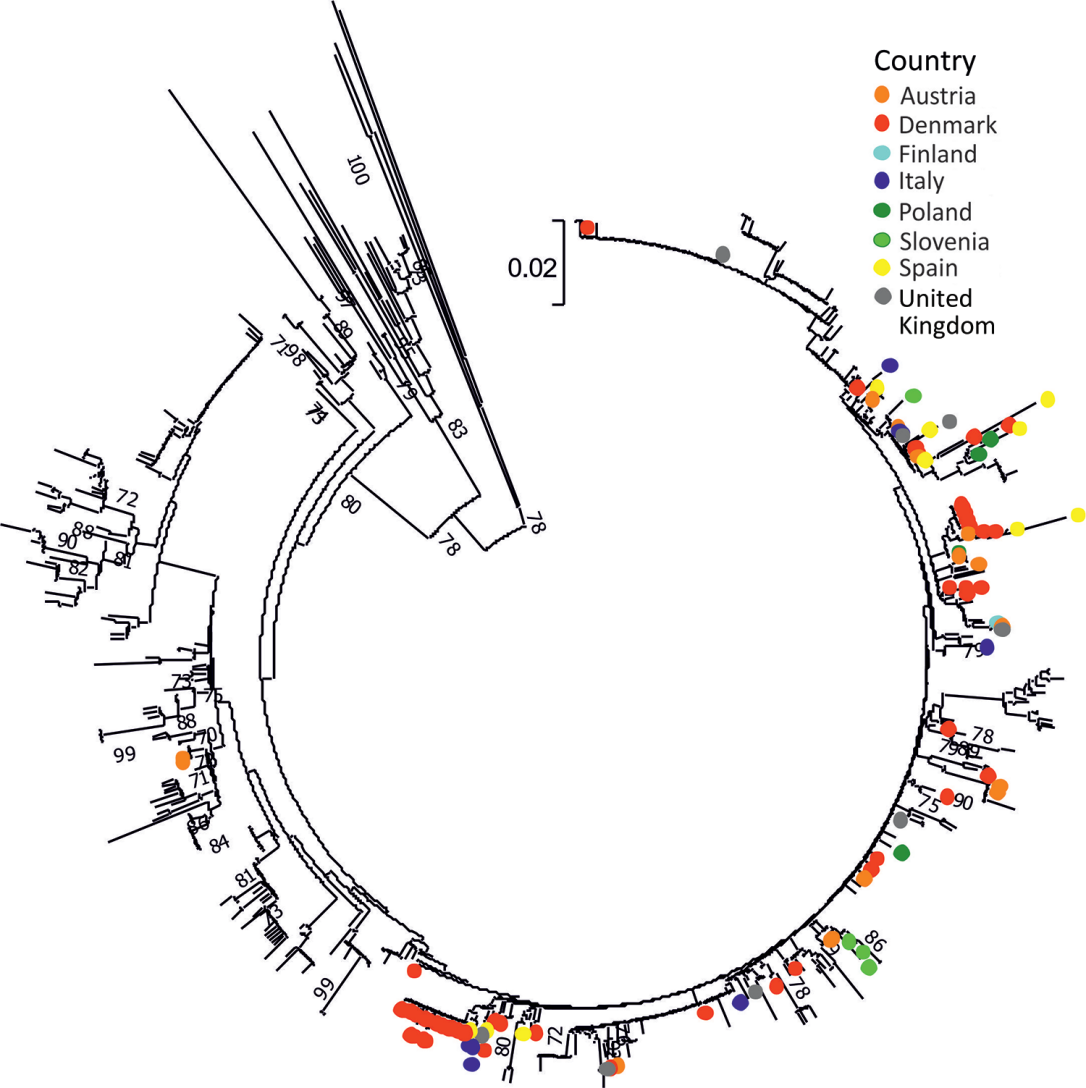
Phylogenetic analysis of the VP3/VP1 region of PeV-3 sequences, Europe, 2015–2021. Neighbor-joining phylogenetic tree of the VP3/VP1 junction region obtained from the study samples (n = 106) is labeled by country of sample origin and compared with 630 available sequences spanning the analyzed region from GenBank. The tree was constructed using MEGA 7 (*17*) using Jukes-Cantor corrected distances, with bootstrap resampling; branches showing 70% or greater supports were labeled. Scale bar indicates substitutions per site. A maximum-likelihood analysis of the same sequence dataset is provided in Appendix Figure 2. PeV, parechovirus type; VP, viral protein.

PeV-A3 was the type most frequently reported by participating laboratories. We performed phylogenetic analysis of 106 available study sequences in the VP3/VP1 junction region to compare relationships between potential country- or region-specific groups of strains and available previously published PeV-A3 variants (Figure 4; Appendix Figure 2). Whereas the resolution of the tree was limited by the relatively short length of sequences analyzed (256 bp), variants from different study regions showed some evidence of clustering, possibly representing local geographic spread (e.g., in Denmark), although there was no evidence for specific variants circulating exclusively in just 1 or a few countries. Numerous separate older lineages of PeV-A3 circulating during 2010–2014 or earlier have largely become extinct (Appendix Figure 2). 

## Discussion 

We report the laboratory capacity, type-related temporal dynamics, epidemiology, and clinical manifestations of parechovirus infections reported from 21 laboratories in 18 countries in Europe over a 7-year study period, 2015–2021. We documented extensive capacity for parechovirus detection in northern, western, and some central European countries participating in our study; no parechovirus testing was reported in Bulgaria, the Czech Republic, the Slovak Republic, Estonia, or Hungary. Those findings were consistent with literature in which limited capacity for parechovirus detection and typing was reported outside western and northern European countries (*18*–*22*).

A total of 1,845 parechovirus infections, most identified through NPEV surveillance systems, were reported by 16 laboratories from 13 countries in Europe that participated in the study. Four national laboratories incorporated parechovirus detection into NPEV passive surveillance, collecting data on positive cases from other laboratories that send samples for sequencing after identifying parechovirus. A similar passive surveillance system, in which laboratories report positive NPEV and parechovirus cases to the Centers for Disease Control and Prevention (CDC), was implemented in the United States (*23*). During 2014–2016 in the United States, ≈100 domestic parechovirus cases were reported to CDC (*24*); in Europe, 540 cases from Finland, the Netherlands, Spain, and England, were reported over a comparable 3-year timeframe, 2015–2017. Those figures highlight the current volume and likely benefits of the data collected in Europe, along with the potential capacity to implement similar systems in additional countries within and beyond our region. 

The capacity for parechovirus testing increased during the study period from ≈9,000 samples tested for parechovirus during 2015–2017 to >14,000 during 2018–2021. Luxemburg, Poland, and Slovenia successfully introduced parechovirus testing in 2017, but some 2018–2021 increases in detection capacity attributable to new data sources were likely offset by several laboratories substantially reducing diagnostic and surveillance testing capacity for pathogens not related to SARS-CoV-2 during the COVID-19 pandemic. The overall detection rate of 1.3% (309/22,928) was lower than previously observed rates of 2%–3% in Denmark (*21*) and 13% in Northern Ireland (*22*). However, it is difficult to compare results from our study with results from studies that focused mainly on select populations, such as children and infants needing intensive care unit admission (*4*). 

Besides countries with passive surveillance, laboratories in 2 countries introduced parechovirus testing for respiratory samples collected during ILI surveillance; because samples were implicitly collected from persons with respiratory symptoms only, persons with other parechovirus symptoms would not have been captured through those means. Although ILI surveillance covered all age groups, young infants were likely underrepresented because only 12/130 parechovirus-positive samples were collected from children <3 months of age, which might explain why most of the parechovirus infections captured through ILI surveillance were identified as PeV-A1, a type uncommon among the youngest infants. Based on this finding, ILI surveillance is less likely to capture PeV-A3 infections in children, especially those <3 months of age, because A3 infection manifests with only respiratory symptoms very rarely. Using only ILI surveillance therefore might not be the best option for identifying parechovirus (*25*). 

Twelve laboratories that reported typing capacity successfully sequenced ≈45% of their positive samples, so 28% of total parechovirus-positive samples reported in this study were typed. PeV-A3, the most common type identified in this study, was mostly associated with neurologic infections in infants <3 months of age. The association of PeV-A3 with severe disease, especially in young children, has been well documented elsewhere (*4*,*5*,*8*,*26*–*29*). Our study confirmed both PeV-A3 detection in infants <3 months of age (77% of all typed cases were from this age group) and its severity of infection (73% of infants <3 months of age manifested neurologic signs). Detection of PeV-A3 in sterile samples, such as CSF and blood, confirms its likely systemic nature, which often leads to severe infection. Most PeV-3 cases were originally identified in even-numbered years (2008, 2010, 2012, 2014, and 2016) in northern Europe, the United States, and Australia (*18*,*19*,*30*,*31*). That biannual seasonal pattern was observed for PeV-A3 in Denmark in spring/summer of 2016–2018, but A3 infections appeared to follow a different 2-year cycle in Spain, with peaks in 2017 and 2019. PeV-A1, on the other hand, appeared to follow an annual cycle peaking later each year. Phylogenetic analysis revealed no notable geographic or seasonal clustering of PeV-A3. 

A 2022 increase in PeV-A3 infections affecting newborns and young infants and often resulting in severe outcomes was noted in the United States using data from its passive surveillance system (*32*–*34*). Those data were used to encourage clinicians to consider parechovirus as a differential diagnosis in cases of fever, sepsis-like syndrome, seizures, or meningitis without another known cause (*32*,*33*). Although our findings demonstrate that passive parechovirus surveillance and diagnostic capacities are already available in Europe, no upsurge in recorded parechovirus infections has been noted to date. In future, better harmonization of data collection could be used to monitor the spread of parechovirus infections across Europe, complement early warning systems, and provide the bases for public health recommendations during upsurges. 

Despite ongoing collection and testing of samples during the COVID-19 pandemic, parechovirus detection frequencies for A3–A6 declined dramatically in 2020–2021 during periods of lockdown, comparable to previously documented decreases observed for enteroviruses, such as enterovirus D68 (*35*). An upsurge in PeV-A1 but not in other types in autumn 2021 mirrored the timing of the reappearance of enterovirus D68 and coincided with the end of COVID-19 lockdown restrictions and increased testing of respiratory samples (*35*). This suggests that PeV-A1 more likely spreads through respiratory routes than other parechovirus types.

In terms of clinical associations, our large-scale description of cases provides evidence for differentiating disease patterns between parechovirus types. A4 and A5 infections were detected largely in infants <3 months of age and more often in sterile samples, such as CSF and blood (Figure 3), both features comparable to previously described epidemiologic and clinical properties of PeV-A3 (*11*,*36*,*37*). Strikingly, parechovirus types A4 and A5 were also primarily detected in children <3 months of age, but PeV-A1 and A6 infections occurred mainly in children 1–5 years of age. 

Fever and a higher frequency of neurologic symptoms were associated with higher percentages of PeV-A3 (44%), A4 (50%), and A5 (30%) than A1 or A6 cases. Further patient characterization is required to evaluate whether PeV-A4 and A5 might be more likely to cause neurologic diseases resembling those from PeV-A3 (*10*,*31*–*33*). Although clinical profiles in our study indicate similar neurologic manifestations for PeV-A3 and A4, another study reported that only 9% of A4 infections resulted in neurologic symptoms, much lower than for A3 (91%) (*10*). It should be noted that almost all PeV-A5 infections in our study were reported by Austria, the Netherlands, and Spain in 2018, and more recently by Italy/Lombardy. Therefore, clinical attributes related to neurologic effects might reflect biologic characteristics of circulating strains rather than differences in parechovirus type.

Collected data were reported as aggregated information, limiting the possibility of calculating risk ratios for associations between specific parechovirus types and clinical symptoms. In addition, each country used different case definitions and criteria for collecting and testing samples. Those limitations should inform interpretation of results and their use as baseline information for future systematic approaches.

In conclusion, we demonstrate that multiple laboratories located in 13 countries in Europe have been collecting and analyzing data on parechovirus infections, including demographic information, clinical features, specimen types, and type sequences. Results of investigating parechovirus epidemiology and collecting and analyzing an increasing amount of data suggest that this virus causes severe infections, especially in very young children. Those findings highlight the need to expand parechovirus diagnostics and typing beyond current participating laboratories and share protocols to develop and initiate more efficient systematic approaches for identifying parechovirus-positive cases in Europe. Future approaches should also include a wider spectrum of age-groups and clinical symptoms. Integrating parechovirus with NPEV surveillance would enable better characterization of parechovirus types and seasonality across and beyond Europe and support outbreak detection to improve clinical and public health awareness and provide resources to limit the spread of parechovirus in Europe. 

Appendix Additional information about a study of parechovirus circulation and surveillance capacity in Europe, 2015–2021.
