# Supplementary material for: Parechovirus A Circulation and Testing Capacities in Europe, 2015–2021
Source: Emerg Infect Dis. 2024 Feb;30(2):234–44. doi: 10.3201/eid3002.230647 (PMC10826775; doi:10.3201/eid3002.230647)
Supplement: Appendix — Additional information about a study of parechovirus circulation and surveillance capacity in Europe, 2015–2021. [file 23-0647-Techapp-s1.pdf]

Article DOI: https://doi.org/10.3201/eid3002.230647

EID cannot ensure accessibility for supplementary materials supplied by authors. Readers who have difficulty accessing supplementary content should contact the authors for assistance.

Parechovirus A Circulation and Testing Capacities in Europe, 2015–2021

Appendix

Appendix Table 1. Countries reported PeV testing details, indicating the name of kit in use or primer and probes information for in-house procedures.

Country (region)^	Institution type	Platform used for PeV-A detection	Primers and probes 5'-3'	Method (ref)
Austria	Hospital virology or microbiology laboratory	Light Mix Molecular Parechovirus (hPeV) TIB Molbiol Cat-No 53-0126-96	Not applicable	Not applicable
Denmark	National PH institute	Various depending on the laboratory identifying the case	Not applicable	Surveillance system (*1*)
Finland	Hospital virology/ microbiology laboratory	In-house multiplex assay for detection of PeV-A, EV, and rhinoviruses	For PeV-A*:<br>Forward: CTGGGGCCAAAAGCCA<br>Reverse: GGTACCTTCTGGGCATCCTTC<br>Probe: FAM-AAACACTAGTTGTAHGGCCC-MGB	(*2*)
Ireland	Diagnostic and virus reference laboratory	In-house	Forward: GGC CCT GAA TGC GGC TAA T<br>Reverse: GGG ATT GTC ACC ATA AGC AGC C<br>Probe: FAM/BHQ-ACC CAA AGT AGT CGG TTC CGC	(*3*)
Italy (Lombardy)	Regional PH institute and academic institution	In-house	Forward: GTAACASWWGCCTCTGGGSCCAAAAG<br>Reverse: GGCCCCWGRTCAGATCCAYAGT<br>Probe: VIC-CCTRYGGGTACCTYCWGGGCATCCTTC	(*4*)
Italy (Lombardy)	Hospital virology/ microbiology laboratory and academic institution	Parechovirus r-gene bioMérieux - Argene	Not applicable	
Italy (Lombardy)	Hospital microbiology	Real-time PCR cycler, Rotor-Gene Q using custom primers and probe	Not reported	(*5*)
Slovenia	Academic institute	BioFire: FilmArray meningitis/encephalitis panel	Not applicable	Not applicable
Spain	National PH institute	Hospitals: Commercial PCR kits: bioMérieux: Realcycler de progenie molecular EV/PeV Filmarray ME, Allplex Seegene meningitis. Enterovirus lab: In-house qRT-PCR for EV/PeV detection	Not applicable	(*6*)
UK (England)	National PH institute	In-house multiplex assay for detection of eEV, PeV-A, and internal control	Forward: GGA TAC CAC GCT YGT GGA YCT TAT GC<br>Reverse: CCC AGR GGC AYC TGT TAC CAG<br>Probe NED-CTT ACT AGA GGA TGG CTG T-MGB.	Not reported
Netherlands	Hospital virology/ microbiology laboratory and	Not reported	Not reported	Not reported

Country (region)^	Institution type	Platform used for PeV-A detection	Primers and probes 5'-3'	Method (ref)
	academic institution			
Norway	National PH institute	In-house	Forward: GTTGTAAGGCCCACGAAGGA<br>Reverse: GRTYTGGCCCACTAGACG<br>Probe: FAM-:<br>AGTGTCNCTTGTTACCTRCGGGTACCTTCT- BHQ1	Not reported
UK (Scotland)	National PH institute	Not reported	Not reported	Not reported
Luxembourg	National PH institute	Not reported	Not reported	Not reported
Slovenia	National PH institute	In-house	Forward: GTAACASWWGCCTCTGGGSCCAAAAG<br>Reverse: GGCCCCWGRTCAGATCCAYAGT<br>Probe: VIC-CCTRYGGGTACCTYCWGGGCATCCTTC	(*4*)
Poland	National PH institute	Not reported	Not reported	Not reported

Appendix Table 2. Nucleotide sequences of PeV-A3 variants analyzed in the study. GenBank accession number available upon request.

Region	Code	Sample year	Sample type
Whole genome (n = 1)	FI-652	2018	Serum
Region 1 (n = 30)			
	NL-02-PEV3	2016	Cerebrospinal fluid
	NL-03-PEV3	2016	Cerebrospinal fluid
	NL-04-PEV3	2016	Cerebrospinal fluid
	NL-05-PEV3	2016	Feces
	NL-06-PEV3	2016	Feces
	NL-07-PEV3	2016	Feces
	NL-08-PEV3	2016	Feces
	NL-09-PEV3	2017	Cerebrospinal fluid
	NL-10-PEV3	2017	Feces
	NL-11-PEV3	2018	Feces
	NL-12-PEV3	2018	Feces
	NL-13-PEV3	2018	Feces
	NL-14-PEV3	2019	Throat swab
	NL-15-PEV3	2019	Feces
	NL-16-PEV3	2019	Feces
	NL-17-PEV3	2019	Feces
	NL-18-PEV3	2019	Feces
	NL-19-PEV3	2019	Feces
	PL_21-2168	2021	Feces
	PL-17-7748	2017	Feces
	PL-19-8606	2019	Feces
	PL-20-38059	2020	Feces
	UK-18-01	2018	Not reported
	UK-18-03	2018	Not reported
	UK-18-04	2018	Not reported
	UK-18-08	2018	Not reported
	UK-18-09	2018	Not reported
	UK-18-11	2018	Not reported
	UK-20-01	2020	Not reported
	UK-20-02	2020	Not reported
Region 2 (n = 105)			
	AT-1021063	2017	Cerebrospinal fluid
	AT-1024103	2017	Cerebrospinal fluid
	AT-1024765	2017	Blood
	AT-1067820	2018	Feces
	AT-1077428	2018	Feces
	AT-1077428	2018	Feces
	AT-1078115	2018	Blood
	AT-1079045	2018	Feces
	AT-1083608	2018	Cerebrospinal fluid
	AT-1089799	2018	Feces
	AT-1089800	2018	Cerebrospinal fluid

Region	Code	Sample year	Sample type
	AT-1090151	2018	Blood
	AT-1090333	2018	Cerebrospinal fluid
	AT-1108562	2018	Feces
	AT-1168350	2019	Feces
	AT-1168521	2019	Blood
	AT-1174412	2019	Cerebrospinal fluid
	DK_012015	2015	Feces
	DK_042016	2016	Feces
	DK_062016	2016	Cerebrospinal fluid
	DK_062016	2016	Feces
	DK_062017	2017	Feces
	DK_062017	2017	Feces
	DK_082016	2016	Feces
	DK_082016	2016	Cerebrospinal fluid
	DK_092016	2016	Feces
	DK_092016	2016	Cerebrospinal fluid
	DK_092016	2016	Feces
	DK_092016	2016	Feces
	DK_092016	2016	Feces
	DK_102016	2016	Feces
	DK_112016	2016	Feces
	DK_112017	2017	Feces
	DK-18-3011	2018	Feces
	DK-18-3051	2018	Feces
	DK-18-3052	2018	Feces
	DK-18-3060	2018	Feces
	DK-18-3061	2018	Feces
	DK-18-3062	2018	Feces
	DK-18-3064	2018	Feces
	DK-18-3065	2018	Cerebrospinal fluid
	DK-18-3066	2018	Feces
	DK-18-30661	2018	Cerebrospinal fluid
	DK-18-3067	2018	Feces
	DK-18-3068	2018	Feces
	DK-18-3069	2018	Feces
	DK-18-3071	2018	Feces
	DK-18-3072	2018	Feces
	DK-18-3073	2018	Feces
	DK-18-3074	2018	Respiratory
	DK-18-3075	2018	Feces
	DK-18-3081	2018	Respiratory
	DK-18-3082	2018	Cerebrospinal fluid
	DK-18-3083	2018	Feces
	DK-18-3084	2018	Cerebrospinal fluid
	DK-18-3085	2018	Feces
	DK-18-3086	2018	Feces
	DK-18-3091	2018	Cerebrospinal fluid
	DK-18-3092	2018	Feces
	DK-18-3093	2018	Feces
	DK-18-3094	2018	Feces
	DK-18-3095	2018	Feces
	DK-18-3096	2018	Cerebrospinal fluid
	DK-18-3097	2018	Feces
	DK-18-3101	2018	Feces
	DK-18-3102	2018	Feces
	DK-18-3103	2018	Respiratory
	DK-18-3104	2018	Feces
	DK-18-3105	2018	Feces
	DK-18-3111	2018	Feces
	DK-18-3112	2018	Feces
	DK-19-3011	2019	Feces
	DK-19-3091	2019	Feces
	DK-19-3121	2019	Feces
	DK-20-3071	2020	Serum
	IT_072017_12	2017	Blood
	IT_112015_14	2015	Respiratory
	IT-082017-13	2017	Respiratory
	IT-19269-18	2018	Feces
	IT-25905-18	2018	Cerebrospinal fluid

Region	Code	Sample year	Sample type
	IT-25906-18	2018	Cerebrospinal fluid
	IT-25907-18	2018	Cerebrospinal fluid
	IT-24432-19	2019	Nasopharyngeal aspirate
	PL_21-2168	2021	Feces
	PL-17-7748	2017	Feces
	PL-19-8606	2019	Feces
	PL-20-38059	2020	Feces
	SP-34858	2018	Blood
	SP-55420	2018	Respiratory
	SP-22796	2019	Respiratory
	SP-26710	2019	Blood
	SP-26785	2019	Respiratory
	SP-29868	2019	Cerebrospinal fluid
	SP-30466	2019	Cerebrospinal fluid
	SP-31527	2019	Feces
	SP-11452	2020	Cerebrospinal fluid
	SP-51159	2021	Respiratory
	UK-18-02	2018	Not reported
	UK-18-03	2018	Not reported
	UK-18-04	2018	Not reported
	UK-18-08	2018	Not reported
	UK-18-09	2018	Not reported
	UK-18-11	2018	Not reported
	UK-20-01	2020	Not reported
	UK-20-02	2020	Not reported
	SI-15558-17	2017	Cerebrospinal fluid
	SI-18695-17	2017	Cerebrospinal fluid
	SI-18761-17	2017	Cerebrospinal fluid
	SI-20963-19	2019	Cerebrospinal fluid

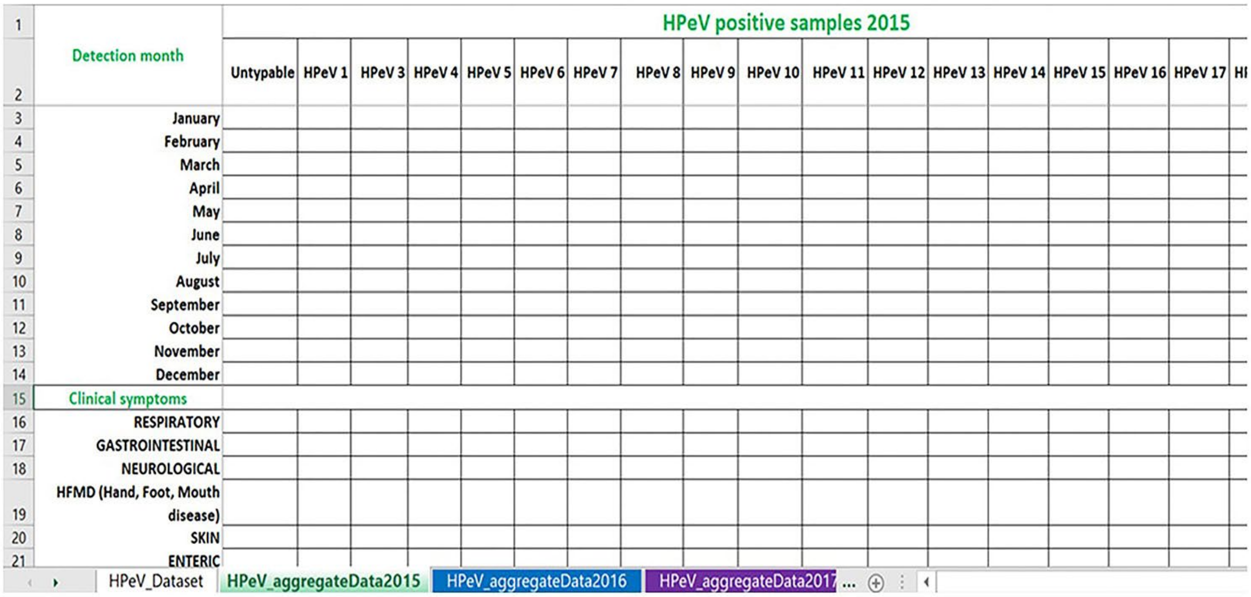

Appendix Figure 1. Structure of data shared with participant laboratories, reporting anonymized aggregated data on number of PeV cases detected by month and/or year, associated clinical symptoms, age group, sample type, sex, and total samples tested for each study year by PeV type. Each structure was replicated for each year during 2015–2021.

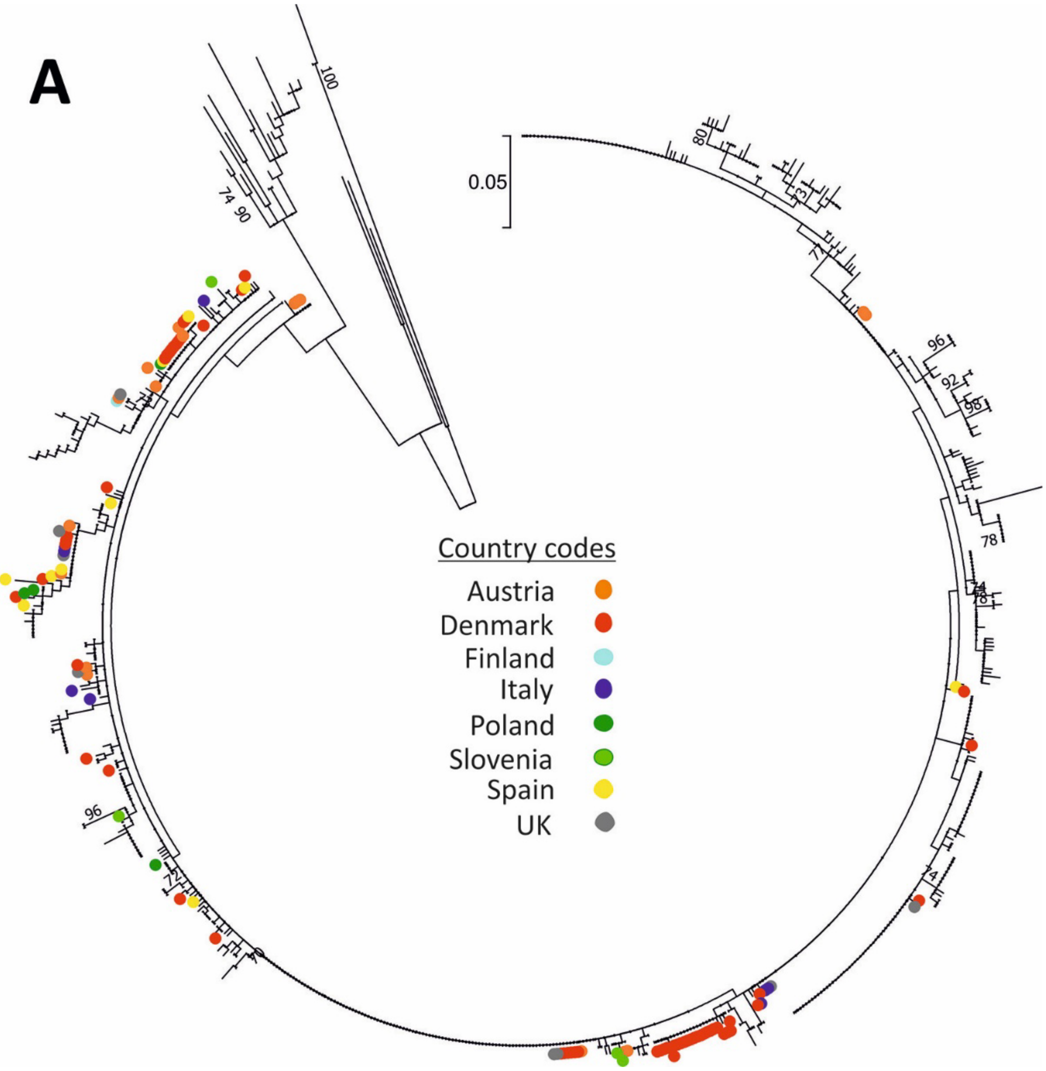

A
0.05
100
74
90
80
71
96
92
98
78
96
Country codes
Austria
Denmark
Finland
Italy
Poland
Slovenia
Spain
UK

B

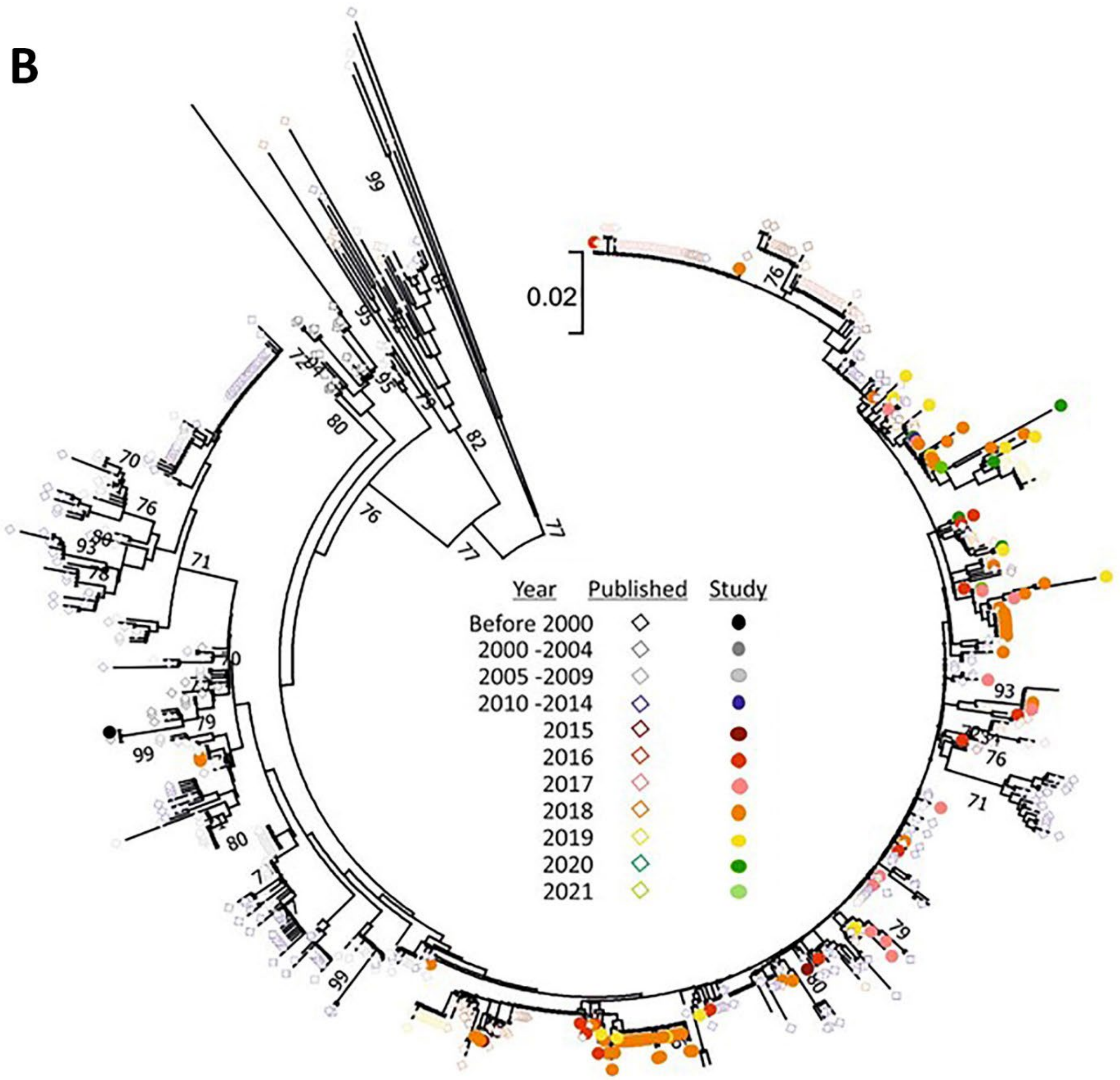

0.02
Year
Published
Study
Before 2000
2000 -2004
2005 -2009
2010 -2014
2015
2016
2017
2018
2019
2020
2021

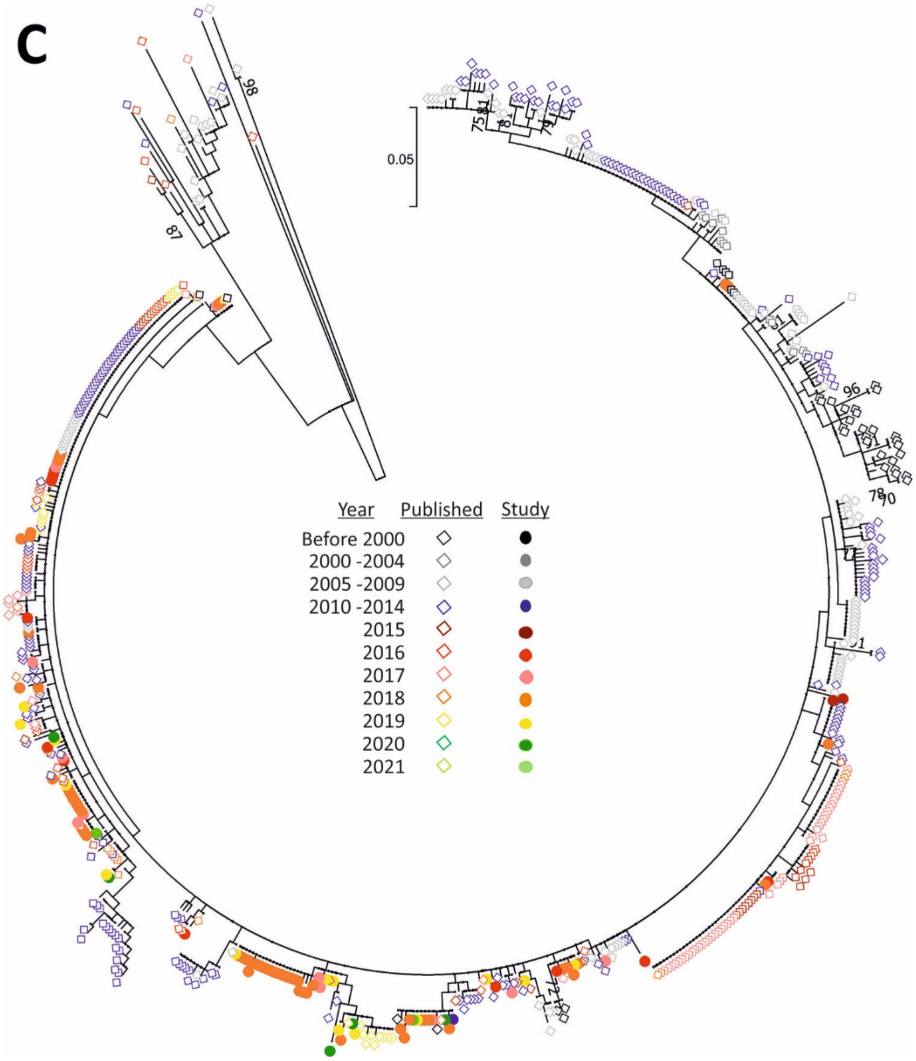

Appendix Figure 2. Phylogenies of the VP3/VP1 junction region for study samples. A) Maximum likelihood phylogeny of the VP3/VP1 junction region for study samples labelled by country of origin, obtained from the study samples with country of origin annotations used for color coding. The tree was constructed using MEGA 7 using Tamura-Nei corrected distances + gamma rate categorization. Replicate trees were generated by bootstrap resampling; we labelled branches showing ≥70% supports. B) Neighbor- joining phylogeny of the VP3/VP1 junction region for study samples and published sequences with sample dates available, obtained from the study samples (n = 106) and 524 sequences with sequence date annotations from GenBank used for color coding. The tree was constructed using MEGA 7 using Jukes-Cantor corrected distances, with bootstrap resampling; we labelled branches showing ≥70% supports. C) Maximum likelihood phylogeny of the VP3/VP1 junction region for study samples and published sequences with sample dates available, obtained from the study samples sequences and available date annotated sequences from with sequence date annotations. The tree was constructed using MEGA 7 using Jukes-Cantor corrected distances, with bootstrap resampling; we labelled branches showing ≥70% supports.

References

1. Fischer TK, Midgley S, Dalgaard C, Nielsen AY. Human parechovirus infection, Denmark. Emerg Infect Dis. 2014;20:83–7. PubMed https://doi.org/10.3201/eid2001.130569

2. Sakthivel SK, Whitaker B, Lu X, Oliveira DB, Stockman LJ, Kamili S, et al. Comparison of fast-track diagnostics respiratory pathogens multiplex real-time RT-PCR assay with in-house singleplex assays for comprehensive detection of human respiratory viruses. J Virol Methods. 2012;185:259–66. PubMed https://doi.org/10.1016/j.jviromet.2012.07.010

3. Corless CE, Guiver M, Borrow R, Edwards-Jones V, Fox AJ, Kaczmarski EB, et al. Development and evaluation of a 'real-time' RT-PCR for the detection of enterovirus and parechovirus RNA in CSF and throat swab samples. J Med Virol. 2002;67:555–62. PubMed https://doi.org/10.1002/jmv.10138

4. Nix WA, Maher K, Johansson ES, Niklasson B, Lindberg AM, Pallansch MA, et al. Detection of all known parechoviruses by real-time PCR. J Clin Microbiol. 2008;46:2519–24. PubMed https://doi.org/10.1128/JCM.00277-08

5. Piralla A, Furione M, Rovida F, Marchi A, Stronati M, Gerna G, et al. Human parechovirus infections in patients admitted to hospital in northern Italy, 2008-2010. J Med Virol. 2012;84:686–90. PubMed https://doi.org/10.1002/jmv.23197

6. Cabrerizo M, Calvo C, Rabella N, Muñoz-Almagro C, del Amo E, Pérez-Ruiz M, et al.; Study Group of Enterovirus and Parechovirus Infections in Children under 3 Years-Old, Spain (PI12-00904). Design and validation of a real-time RT-PCR for the simultaneous detection of enteroviruses and parechoviruses in clinical samples. J Virol Methods. 2014;208:125–8. PubMed https://doi.org/10.1016/j.jviromet.2014.08.008
